# Acidified drinking water attenuates motor deficits and brain pathology in a mouse model of a childhood neurodegenerative disorder

**DOI:** 10.1038/s41598-022-12981-0

**Published:** 2022-05-30

**Authors:** Attila D. Kovács, Logan M. Langin, Jose L. Gonzalez Hernandez, David A. Pearce

**Affiliations:** 1 Pediatrics and Rare Diseases Group, Sanford Research, 2301 E. 60th Street N., South Dakota, Sioux Falls, 57104 USA; 2 Department of Pediatrics, Sanford School of Medicine, University of South Dakota, Sioux Falls, SD USA; 3 Department of Agronomy, Horticulture, and Plant Science, South Dakota State University, Brookings, SD USA; 4 Department of Biology and Microbiology, South Dakota State University, Brookings, SD USA

**Keywords:** Neuroscience, Diseases of the nervous system, Genetics of the nervous system

## Abstract

We recently demonstrated that HCl-acidified drinking water, which is widely used in laboratory animal facilities, had some beneficial effects in the *Cln3*^*−/−*^ mouse model of juvenile Batten disease, a neurodegenerative lysosomal storage disorder^1^. Here we tested if acidified drinking water has therapeutic effects in *Cln1*^*R151X*^ nonsense mutant mice, a model of the infantile form of Batten disease. In *Cln1*^*R151X*^ mice, acidified drinking water received from weaning prevented the impairment in pole climbing ability measured at 3 and 6 months of age. Histopathological analysis of the brain at 6 months showed that acidified drinking water decreased the amount of lysosomal storage material, reduced astrocytosis in the striatum and somatosensory barrelfield cortex, and attenuated microglial activation in the thalamus. Compared to wild-type mice, the gut microbiota of *Cln1*^*R151X*^ mice was markedly different. Acidified drinking water significantly altered the gut microbiota composition of *Cln1*^*R151X*^ mice, indicating a contribution of gut bacteria to the therapeutic effects of acidified water. Our results in *Cln1*^*R151X*^ mice suggest that acidified drinking water may have beneficial effects for patients with infantile Batten disease. This study also verifies that acidified drinking water can modify disease phenotypes in mouse models, contributing to the inter-laboratory variations in neurological and pathological findings.

## Introduction

Batten disease is a group of fatal, mostly pediatric lysosomal storage disorders characterized by progressive neurodegeneration. The different forms of Batten disease are caused by mutations in one of the 13 *CLN* genes (*CLN1-14*; *CLN9* was a mistake). The 13 *CLN* genes encode rather different proteins including soluble lysosomal enzymes (*CLN1-2, 10, 13*), transmembrane proteins of the lysosome and endoplasmic reticulum (*CLN3,6–8*), a P-type ATPase (*CLN12*) and a K^+^ channel-associated protein (*CLN14*)^2^. Mutations in the *CLN1* gene cause classic infantile CLN1 Batten diseases^2^. The *CLN1* gene encodes palmitoyl protein thioesterase 1, a soluble lysosomal enzyme that removes long-chain fatty acids linked to cysteine residues of proteins^3^. Infantile CLN1 disease begins at 1–2 years of age causing developmental delay and decelerated cranial growth. The clinical presentation includes decreased muscle tone, microcephaly, progressive loss of vision, and epilepsy. Patients are in a vegetative state for years before dying at around the age of 10^4^. No treatment is currently available for infantile CLN1 disease. We generated a new mouse model of the disease: *Cln1*^*R151X*^ mice that carry a frequent disease-causing human nonsense mutation^5^. A nonsense mutation results in a premature stop codon. During translation the premature stop codon is recognized and the mutant mRNA is degraded by the nonsense-mediated decay pathway to prevent the generation of a potentially harmful, truncated protein^6^. Accordingly, in *Cln1*^*R151X*^ mice, *Cln1* mRNA level and the *Cln1* gene product, palmitoyl protein thioesterase 1, are both profoundly decreased^5^. In the brain of *Cln1*^*R151X*^ mice, similarly to patients with infantile CLN1 Batten disease, autofluorescent storage material accumulates and widespread activation of astrocytes and microglial cells occur^5^. *Cln1*^*R151X*^ mice also have motor deficits as measured by a modified vertical pole test and the rotarod test at 3 and 5 months of age^5^. *Cln1*^*R151X*^ mice prematurely die around 8 months of age.

In many laboratory animal facilities including the Jackson Laboratory e.g., the drinking water is acidified with HCl to a pH between 2.5 and 3.0 to prevent the spread of pathogenic bacteria. The assumption is that acidified drinking water has no physiological effects. We have, however, recently demonstrated in the *Cln3*^*−/−*^ mouse model of juvenile CLN3 Batten disease that acidified drinking water temporarily attenuated the motor deficits, had beneficial effects on certain behavioral parameters, and prevented microglial activation and attenuated astrocytosis in the brain. These effects of acidified water were accompanied by a few specific changes in the gut microbiota of *Cln3*^*−/−*^ mice^1^.

In the present study, we tested if acidified drinking water has therapeutic effects in the *Cln1*^*R151X*^ nonsense mutant mouse model of infantile CLN1 Batten disease. Specifically, we examined if acidified drinking water administered from weaning (postnatal day 21) affects the motor deficits of *Cln1*^*R151X*^ mice at an early (3 months) and a late stage (6 months) of the disease, as measured in a modified vertical pole test and an accelerating rotarod test. The effects of acidified drinking water on the neuropatholological changes (accumulation of autofluorescent lysosomal storage material, activation of astrocytes and microglial cells) in the brain of 6-month-old *Cln1*^*R151X*^ mice were also assessed. We also examined how acidified water altered the gut microbiota composition of *Cln1*^*R151X*^ and wild-type mice at 3 and 6 months of age, and if specific changes in the gut microbiota correlate with the therapeutic effects of acidified water.

In this study, we used male mice only for the following reasons. We have previously found using the *Cln3*^*−/−*^ and *Cln3*^*Δex7/8*^ mouse models of juvenile Batten disease that *Cln3*^*−/−*^ and *Cln3*^*Δex7/8*^ males have more and more severe neurological phenotypes than females and thus, are more suitable for therapeutic studies^7^. Therefore, since the *Cln1*^*R151X*^ nonsense mutant mouse model was generated for testing new therapies including nonsense suppressive drugs, during the characterization of *Cln1*^*R151X*^ mice we only examined the neurological phenotypes and neuropathological changes in male mice^5^.

## Results

### Acidified drinking water in ***Cln1***^***R151X***^ mice prevents the impairment in pole climbing ability as measured at 3 and 6 months of age

**Figure 1:**
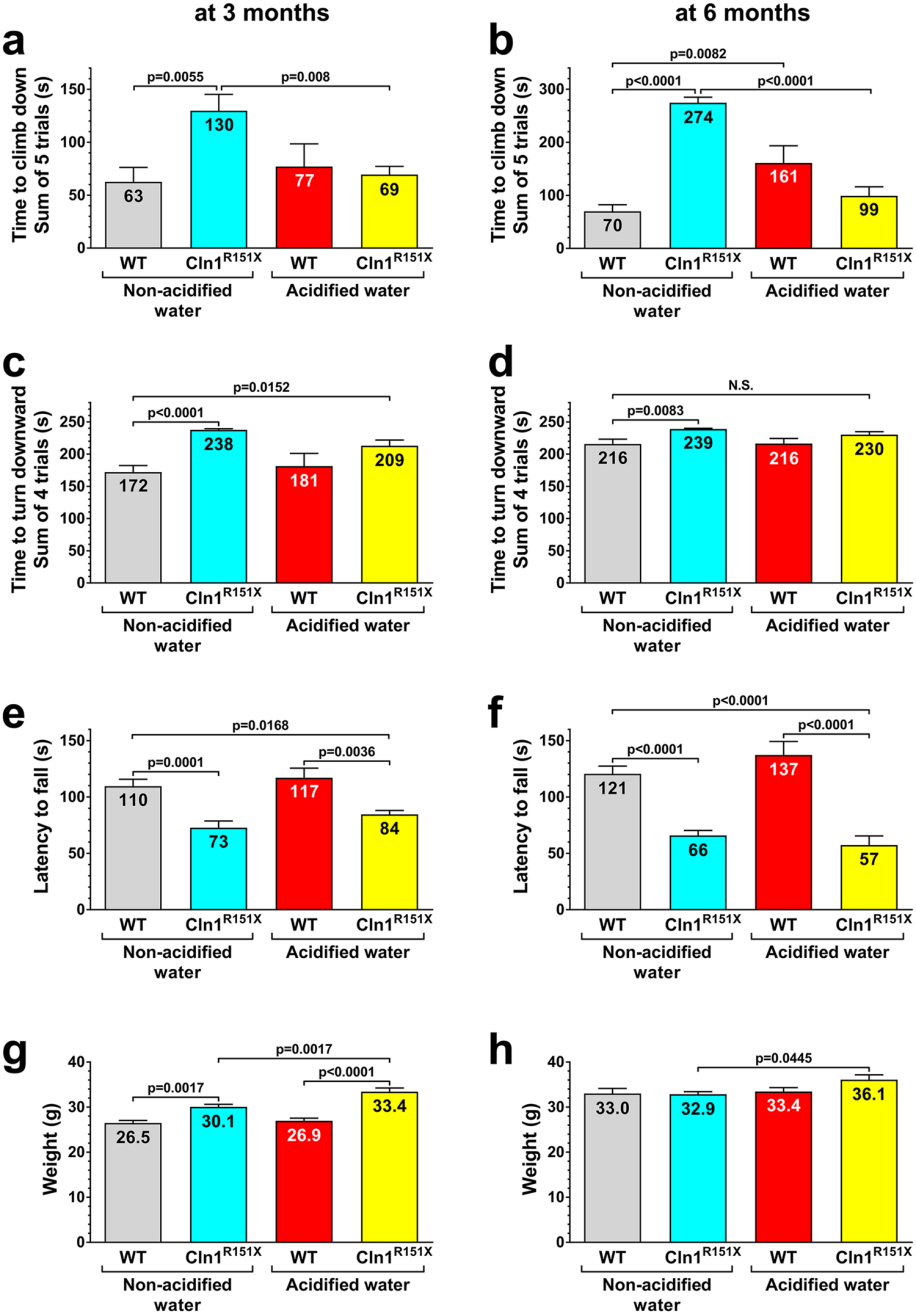
Acidified drinking water in *Cln1*^*R151X*^ mice prevents the impairment in pole climbing ability. A group of *Cln1*^*R151X*^ and wild-type (WT) male mice received acidified drinking water from weaning (21 days of age) and were compared to male mice that always had non-acidified drinking water. At 3 and 6 months of the age, a vertical pole was used to test the climb down (**a**,**b**) and turn downward (**c**,**d**) ability of mice. One day later, the same mice were also tested on an accelerating rotarod (from 0 to 48 rpm in 240 s) (**e**,**f**). (**g**,**h**) Weight of the mice at 3 and 6 months. Acidified drinking water in *Cln1*^*R151X*^ mice prevented the impairment in pole climbing ability at 3 and 6 months of age (**a**,**b**). Acidified drinking water decreased the time to turn downward at both 3 and 6 months but the differences were not statistical significant (**c**,**d**). In line with our previous results^1^, acidified drinking water impaired the pole-descending ability of WT mice at 6 months (**b**). In the accelerating rotarod test, which measures motor coordination and balance, *Cln1*^*R151X*^ mice receiving non-acidified drinking water performed poorly at both 3 and 6 months, and acidified water apparently had no effect on their rotarod performance (**e**,**f**). However, 6-month-old *Cln1*^*R151X*^ mice that received acidified drinking water displayed a strange behavior in the rotarod test: they did not want to stay on the rod of the rotarod even when the rod was not moving. Therefore, their rotarod performance was confounded by their unwillingness to stay on the rod. The movement and home cage activity of these mice were normal (as observed by the experimenter during the behavioral tests and by animal care technicians daily in the vivarium), and these mice performed similarly to WT mice in the pole descending task (**a**,**b**). Columns and bars represent mean ± SEM (WT and *Cln1*^*R151X*^ on non-acidified water: 14 and 19 mice, respectively; WT and *Cln1*^*R151X*^ on acidified water: 10 and 18 mice, respectively). Two-way ANOVA analysis showed a significant interaction between the two factors, genotype and type of drinking water, for the time to climb down the pole at both 3 and 6 months (p = 0.0154 and p < 0.0001), and for weight at 3 months (p = 0.0465). Only the genotype had a significant effect on the time to turn downward on the pole (p < 0.0001 at 3 months, p = 0.0008 at 6 months), and on the rotarod test results (p < 0.0001 at both 3 and 6 months). In two-way ANOVA, Tukey’s post-test was used to calculate statistical significance in multiple comparisons. *NS* not significant (**d**).

*Cln1*^*R151X*^ mice display motor deficits in a modified vertical pole test and the rotarod test^5,7^. To investigate if HCl-acidified drinking water (average pH: 2.8) has therapeutic effects in *Cln1*^*R151X*^ mice, *Cln1*^*R151X*^ and wild-type (WT) mice received acidified drinking water starting at 21 days of age (weaning), and their motor function was assessed at 3 and 6 months of age. Six months represent a late stage of the disease since *Cln1*^*R151X*^ mice prematurely die around 8 months of age. In the modified vertical pole test, *Cln1*^*R151X*^ mice receiving non-acidified drinking water had difficulties to climb down the pole, and acidified drinking water restored their pole-climbing ability to the WT level at both 3 and 6 months of age (Fig. 1a,b). The time to turn downward at the top of the pole was another parameter measured in the modified vertical pole test. It took much longer for *Cln1*^*R151X*^ mice receiving non-acidified drinking water to turn downward as compared to WT mice (Fig. 1c,d). Acidified drinking water decreased the time to turn downward at both 3 and 6 months but the differences were not statistically significant (Fig. 1c,d). In line with our previous results^1^, acidified drinking water impaired the pole-descending ability of WT mice at 6 months (Fig. 1b).

In the accelerating rotarod test, which measures motor coordination and balance, *Cln1*^*R151X*^ mice receiving non-acidified drinking water performed poorly at both 3 and 6 months, and acidified water apparently had no effect on their rotarod performance (Fig. 1e,f). However, 6-month-old *Cln1*^*R151X*^ mice that received acidified drinking water displayed a strange behavior in the rotarod test: they did not want to stay on the rod of the rotarod even when the rod was not moving. It was very difficult to have all mice staying on the rod and start the test. Therefore, the rotarod performance of these mice was confounded by their unwillingness to stay on the rod. The movement and home cage activity of these mice were normal (as observed by the experimenter during the behavioral tests and by animal care technicians daily in the vivarium), and these mice performed similarly to WT mice in the pole descending task (Fig. 1a,b).

We also collected weight data and found that *Cln1*^*R151X*^ mice receiving non-acidified or acidified drinking water were heavier than WT mice at 3 months of age but these weight differences disappeared by 6 months (Fig. 1g,h). Furthermore, *Cln1*^*R151X*^ mice on acidified drinking water were significantly heavier than *Cln1*^*R151X*^ mice on non-acidified drinking water at both 3 and 6 months of age (Fig. 1g,h). Correlation analysis revealed that in the modified vertical pole test, the climb down time did not correlate with the weight at 3 or 6 months. The time to turn downward on the pole however, showed a positive correlation with the weight at 3 months but not at 6 months (at 3 months, heavier mice turned downward in a longer time; p = 0.0002). In the rotarod test, at both 3 and 6 months, the latency to fall negatively correlated with the weight (p < 0.002; heavier mice fell sooner). All in all, the higher weight of *Cln1*^*R151X*^ mice receiving acidified drinking water negatively affected their performance in turning downward on the vertical pole and in the rotarod test. In the case of 3-month-old *Cln1*^*R151X*^ mice that received non-acidified drinking water, the increased body weight might contribute to the poor rotarod performance and the deficit in turning downward on the pole.

Motor learning, due to repeated test sessions, contributes to the rotarod performance. To assess motor learning, we compared the data of the 3 rotarod test sessions using repeated measures 2-way ANOVA with treatment group and test session as the two factors, and with Tukey’s post-test for multiple comparisons. At 3 months of age, WT mice receiving non-acidified or acidified drinking water did not show statistically significant motor learning. In contrast, 3-month-old *Cln1*^*R151X*^ mice displayed effective motor learning: improving in the 2nd (p = 0.0004) and further improving in the 3rd test session (p = 0.0183) when received non-acidified water, and improving only in the 2nd test session (p < 0.0001) when received acidified water. At 6 months of age, both WT and *Cln1*^*R151X*^ mice showed effective motor learning. WT mice on non-acidified or acidified water improved their performance in the 2nd test session (p < 0.0274). Six-month-old *Cln1*^*R151X*^ mice improved their rotarod performance in the 3rd test session (p = 0.0115) when received non-acidified water, and in the 2nd test session (p = 0.0405) when received acidified water. Despite their motor learning capability, the motor coordination and/or balance of *Cln1*^*R151X*^ mice were severely impaired since they fell from the rotating rod significantly sooner than WT mice (see Fig. 1e,f).

### Acidified drinking water in *Cln1*^*R151X*^ mice decreases the amount of lysosomal storage material in the brain, reduces astrocytosis in the striatum and somatosensory barrelfield cortex, and attenuates microglial activation in the thalamus

**Figure 2:**
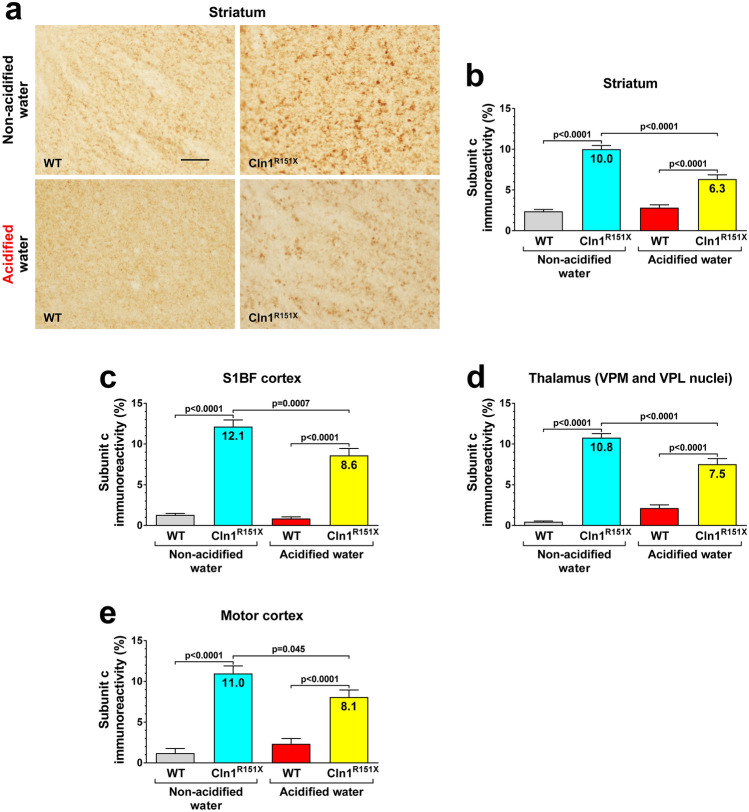
Acidified drinking water decreases the amount of lysosomal storage material in the brain of *Cln1*^*R151X*^ mice. A group of *Cln1*^*R151X*^ and wild-type (WT) male mice received acidified drinking water from weaning (21 days of age) and were compared to male mice that always had non-acidified drinking water. At 6 months of age, lysosomal storage material was measured in the brain using immunohistochemical staining for subunit c of the mitochondrial ATP synthase, a common constituent of the storage material in different forms of Batten disease. Quantitative image analysis was performed in two sensory area, the somatosensory barrel field (S1BF) cortex and the ventral posteromedial (VPM)/ventral posterolateral (VPL) nuclei of the thalamus, and in two regions responsible for motor control, the motor cortex and striatum. (**a**,**b**) Striatum. (**c**) S1BF cortex. (**d**) Thalamus. (**e**) Motor cortex. The graphs show percent area of subunit c immunoreactivity. Columns and bars represent mean ± SEM (24–96 fields from 3–4 mice in each experimental group). Two-way ANOVA analysis showed a significant interaction between the two factors, genotype and type of drinking water, in all four brain regions (Striatum: p < 0.0001; S1BF cortex: p = 0.0201; Thalamus: p < 0.0001; Motor cortex: p = 0.0131). In two-way ANOVA, Tukey’s post-test was used to calculate statistical significance in multiple comparisons. The size of the scale bar in the image is 50 µm.

**Figure 3:**
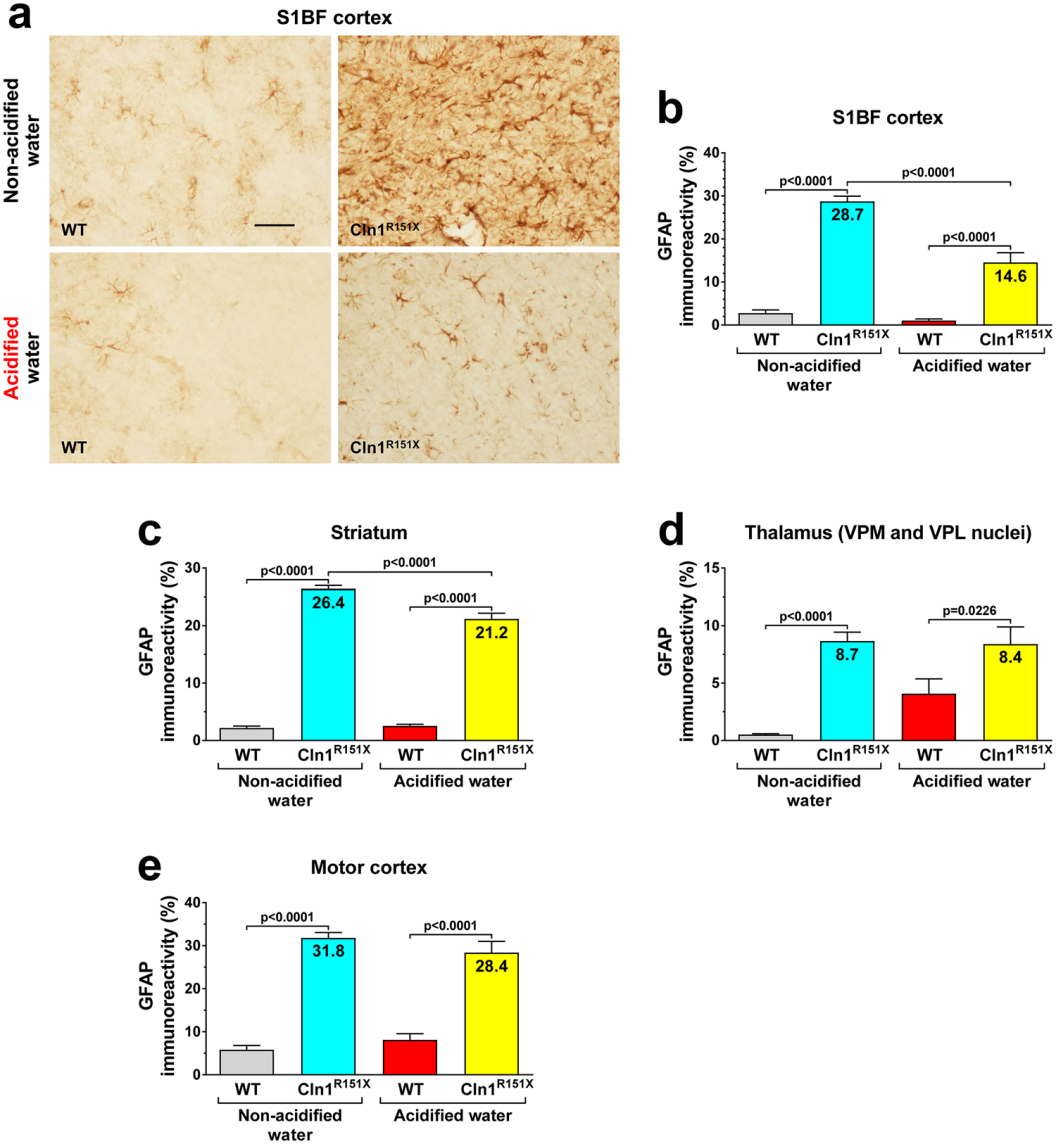
Acidified drinking water reduces astrocytosis in the striatum and somatosensory barrelfield cortex of *Cln1*^*R151X*^ mice. A group of *Cln1*^*R151X*^ and wild-type (WT) male mice received acidified drinking water from weaning (21 days of age) and were compared to male mice that always had non-acidified drinking water. At 6 months of age, astrocytic activation was assessed by immunostaining for the glial fibrillary acidic protein (GFAP) that forms intermediate filaments. Quantitative image analysis was performed in two sensory area, the somatosensory barrel field (S1BF) cortex and the ventral posteromedial (VPM)/ventral posterolateral (VPL) nuclei of the thalamus, and in two regions responsible for motor control, the motor cortex and striatum. (**a**,**b**) S1BF cortex. (**c**) Striatum. (**d**) Thalamus. (**e**) Motor cortex. The graphs show percent area of GFAP immunoreactivity. Columns and bars represent mean ± SEM (28–120 fields from 3–6 mice in each experimental group). Two-way ANOVA analysis showed a significant interaction between the two factors, genotype and type of drinking water, in the S1BF cortex and striatum (p < 0.0001). Only the genotype had a significant effect in the thalamus and motor cortex (p < 0.0001). In two-way ANOVA, Tukey’s post-test was used to calculate statistical significance in multiple comparisons. The size of the scale bar in the image is 50 µm.

**Figure 4:**
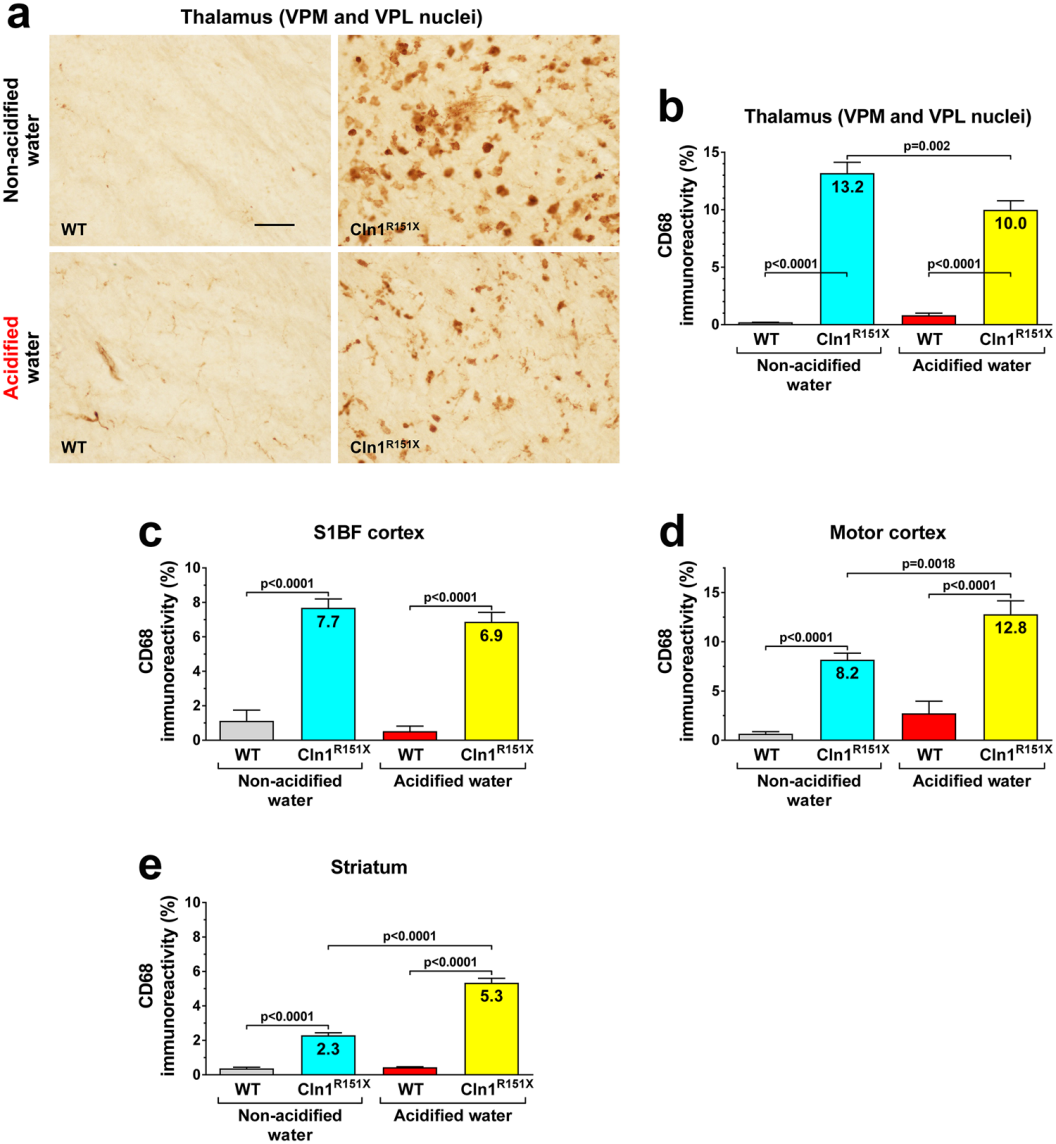
Acidified drinking water in *Cln1*^*R151X*^ mice attenuates microglial activation in the thalamus and augments it in the motor cortex and striatum. A group of *Cln1*^*R151X*^ and wild-type (WT) male mice received acidified drinking water from weaning (21 days of age) and were compared to male mice that always had non-acidified drinking water. At 6 months of age, microglial activation was measured by immunostaining for CD68, a marker of activated microglia. Quantitative image analysis was performed in two sensory area, the somatosensory barrel field (S1BF) cortex and the ventral posteromedial (VPM)/ventral posterolateral (VPL) nuclei of the thalamus, and in two regions responsible for motor control, the motor cortex and striatum. (**a**,**b**) Thalamus. (**c**) S1BF cortex. (**d**) Motor cortex. (**e**) Striatum. The graphs show percent area of CD68 immunoreactivity. Columns and bars represent mean ± SEM (32–108 fields from 3 to 6 mice in each experimental group). Two-way ANOVA analysis showed a significant interaction between the two factors, genotype and type of drinking water, in the thalamus and striatum (p = 0.0026 and p < 0.0001). Both the genotype and type of drinking water, without a significant interaction, affected astrocytosis in the motor cortex (p < 0.0001 and p = 0.0002), and only the genotype had a significant effect in the S1BF cortex (p < 0.0001). In two-way ANOVA, Tukey’s post-test was used to calculate statistical significance in multiple comparisons. The size of the scale bar in the image is 50 µm.

*Cln1*^*R151X*^ mice display characteristic neuropathological changes that include accumulation of lysosomal storage material and astrocytic and microglial activation in the brain^5^. These changes were measured at 6 months of age in two sensory area, the somatosensory barrel field (S1BF) cortex and the ventral posteromedial (VPM)/ventral posterolateral (VPL) nuclei of the thalamus, and in two regions responsible for motor control, the motor cortex and striatum. Lysosomal storage material was measured in the brain using immunohistochemical staining for subunit c of the mitochondrial ATP synthase, a common constituent of the storage material in different forms of Batten disease^8^. Acidified drinking water markedly decreased the amount of accumulated storage material in all brain regions examined (Fig. 2). Astrocytic activation was assessed by immunostaining for the glial fibrillary acidic protein (GFAP) that forms intermediate filaments. Acidified drinking water significantly reduced astrocytosis in the S1BF cortex and the striatum, whereas it did not affect astrocytic activation in the motor cortex and thalamus (Fig. 3). Microglial activation was measured by immunostaining for CD68, a marker of activated microglia. While acidified drinking water attenuated microglial activation in the thalamus of *Cln1*^*R151X*^ mice, it increased CD68 immunoreactivity in the motor cortex and the striatum (Fig. 4). Acidified drinking water had no effect on microglial activation in the S1BF cortex (Fig. 4c). Since astrocytes and microglial cells in the mammalian central nervous system display region-specific molecular and functional diversity^9,10^, it is not surprising that acidified water had distinct effects in different brain regions.

### *Cln1*^*R151X*^ and wild-type mice have different gut microbiota and acidified drinking water alters them differently

**Figure 5:**
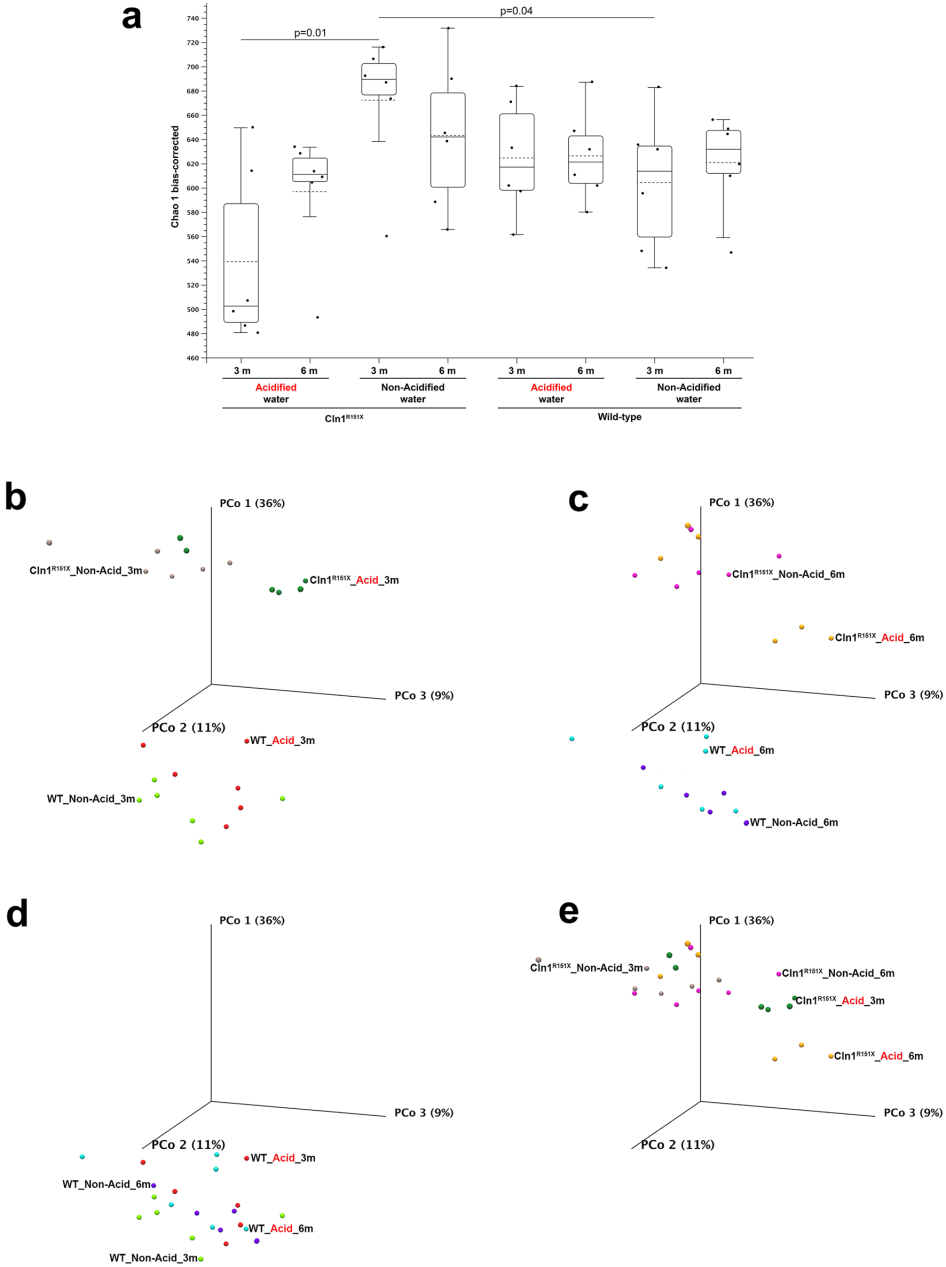
The gut microbiota compositions of *Cln1*^*R151X*^ and wild-type mice are significantly different and acidified water differentially changes them. A group of *Cln1*^*R151X*^ and wild-type (WT) male mice received acidified drinking water from weaning (21 days of age) and were compared to male mice that always had non-acidified drinking water. Fecal pellets were collected at 3 and 6 months of age to analyze the gut microbiota by 16S rRNA gene sequencing. (**a**) Alpha diversity, the within-sample taxonomical diversity, was quantified by the Chao 1 bias-corrected diversity index. At 3 months of age, on non-acidified water, the alpha diversity in *Cln1*^*R151X*^ mice was significantly higher than in WT mice. Acidified drinking water markedly reduced alpha diversity in *Cln1*^*R151X*^ mice at 3 months, but at 6 months, the difference between acidified and non-acidified water became statistically insignificant. Box and whisker plot: the black dots show the individual data (n = 6 mice, from 3–5 different cages for each group). Statistical significance was determined by a Kruskal–Wallis H test to assess if all the groups follow the same distribution, and a Mann–Whitney U test to determine which pairs of groups follow different distributions. (**b**,**e**) Beta diversity, the measure of dissimilarity among the different groups, was determined by principal coordinate analysis. Each symbol represent an individual mouse (n = 6 mice, from 3–5 different cages for each group). Principal coordinate analysis showed markedly different clustering of the gut microbial community in *Cln1*^*R151X*^ and WT mice at 3 and 6 months of age on both acidified and non-acidified water (**b**,**c**). Acidified drinking water in *Cln1*^*R151X*^ mice caused a pronounced change in the global microbiota composition at both 3 and 6 months (p = 0.00433 and 0.04329), whereas, in WT mice, it only altered the bacterial community structure at 6 months (p = 0.02165) (**b**,**c**). Aging (3 vs. 6 months) did not cause statistically significant changes in beta diversity in *Cln1*^*R151X*^ and WT mice on either non-acidified or acidified water (**d**,**e**). Statistical significance in beta diversity (Bray–Curtis) was determined by PERMANOVA analysis.

Through the gut-brain axis and by secreted metabolites acting in the central nervous system, the gut microbiota affects brain function. Therefore, we analyzed the gut bacterial composition of *Cln1*^*R151X*^ and WT mice by sequencing the 16S ribosomal RNA gene in fecal pellets preserved during the pole climbing test. The Chao 1 bias-corrected diversity index was used to quantify alpha diversity, the within-sample taxonomical diversity. At 3 months of age, on non-acidified water, the alpha diversity in *Cln1*^*R151X*^ mice was significantly higher than in WT mice (Fig. 5a). Acidified drinking water markedly reduced alpha diversity in *Cln1*^*R151X*^ mice at 3 months, but at 6 months, the difference between acidified and non-acidified water became statistically insignificant (Fig. 5a). Beta diversity, the measure of dissimilarity among the different groups, was determined by principal coordinate analysis. The gut microbial community in *Cln1*^*R151X*^ and WT mice at 3 and 6 months of age on both acidified and non-acidified water showed markedly different clustering (Fig. 5b,c). Acidified drinking water in *Cln1*^*R151X*^ mice caused a pronounced change in the global microbiota composition at both 3 and 6 months (p = 0.00433 and 0.04329), whereas, in WT mice, it only altered the bacterial community structure at 6 months (p = 0.02165) (Fig. 5b,c). The altered gut microbiota of *Cln1*^*R151X*^ mice receiving acidified drinking water was still different from the gut microbiota of WT mice on non-acidified water at both 3 and 6 months (p = 0.00216). Aging (3 vs. 6 months) did not cause statistically significant changes in beta diversity in *Cln1*^*R151X*^ and WT mice on either non-acidified or acidified water (Fig. 5d,e).

At the individual taxonomic ranks, acidified drinking water had major but differential effects in *Cln1*^*R151X*^ and WT mice (Supplementary Table S1, Figs. S1–S5). Fold differences in Supplementary Table S1 were calculated using the raw abundances. At the phylum level, acidified drinking water in *Cln1*^*R151X*^ mice caused a marked decrease in the abundance of *Deferribacteres* at 3 months (− 37 fold) and of *Verrucomicrobia* at 3 and 6 months (− 821 and − 346 fold) (Supplementary Table S1). In wild-type mice, acidified drinking water increased the abundance of the *Tenericutes* phylum by tenfold at 3 months (Supplementary Table S1). In *Cln1*^*R151X*^ mice, acidified water significantly changed the abundance of 4 bacterial classes at 3 months (*Deferribacteres*, *Saccharimonadia*, *Alphaproteobacteria*, *Verrucomicrobiae*) and one class at 6 months (*Verrucomicrobiae*), 3 orders at 3 months (*Deferribacterales*, *Clostridiales*, *Verrucomicrobiales*) and one order at 6 months (*Verrucomicrobiales*), 8 families at 3 months (*Akkermansiaceae*, *Muribaculaceae*, *Prevotellaceae*, *Deferribacteraceae*, *Rikenellaceae*, *Enterococcaceae*, *Marinifilaceae*, *Erysipelotrichaceae*) and 2 families at 6 months (*Akkermansiaceae*, *Bifidobacteriaceae*) (Supplementary Table S1). In WT mice, acidified water significantly altered the abundance of 2 classes at 3 months (*Alphaproteobacteria*, *Mollicutes*) and one class at 6 months (*Mollicutes*), 2 orders at 3 months (*Rickettsiales*, *Anaeroplasmatales*), and one family at 3 months (*Anaeroplasmataceae*) (Supplementary Table S1).

At the genus level, acidified drinking water markedly changed the abundance of *Odoribacter*, *Uncultured bacterium-2*, *Prevotellaceae UCG-001*, *Alistipes*, *Mucispirillum*, *Enterococcus*, *Family XIII UCG-001*, *Lachnospiraceae UCG-004* and *UCG-006*, *Marvinbryantia*, *Intestinimonas*, *Ruminiclostridium 5*, *Ruminococcaceae UCG-009* and *UCG-013*, *Faecalibaculum*, *Bilophila*, *Uncultured-8* and *Akkermansia* at 3 months, and of *Bifidobacterium*, *Lachnospiraceae NK4A136 group*, *Marvinbryantia*, *Ruminococcaceae UCG-013*, *Desulfovibrio*, *Uncultured-8* and *Akkermansia* at 6 months (Supplementary Table S1). Acidified water in WT mice only altered the abundance of the *Anaeroplasma* genus by 3 months of age, and of the *A2*, *Lachnospiraceae UCG-001* and *Ruminococcaceae UCG-005* genera by 6 months of age (Supplementary Table S1).

While significant age-dependent changes (3 vs. 6 months) were observed in the gut microbiota at the phylum, class, order, family and genus levels in *Cln1*^*R151X*^ mice receiving non-acidified drinking water, age-dependent changes in *Cln1*^*R151X*^ mice receiving acidified water were completely absent (Supplementary Table S1). WT mice on non-acidified water displayed age-dependent changes in their gut microbiota at the phylum and genus levels, whereas in WT mice receiving acidified water age-dependent changes only occurred at the genus level (Supplementary Table S1).

## Discussion

In this study, we demonstrated that acidified drinking water had beneficial effects in *Cln1*^*R151X*^ mice, a mouse model of the fatal childhood neurodegenerative disorder, infantile CLN1 disease. Acidified water received from 21 days of age decreased the amount of lysosomal storage material in every brain region examined, reduced astrocytosis in the striatum and somatosensory barrelfield cortex, attenuated microglial activation in the thalamus, and preserved the ability of *Cln1*^*R151X*^ mice to climb down a vertical pole as quickly and proficiently as wild-type mice.

Preservation of the neurological function, however, was task-specific. In an accelerating rotarod test, *Cln1*^*R151X*^ mice receiving acidified drinking water performed poorly, and displayed a strange, rotarod-specific behavior at 6 months of age: they did not want to stay on the still rod, making it difficult to start and carry out the test. Only 6-month-old *Cln1*^*R151X*^ mice on acidified drinking water displayed this behavior. The movement and home cage activity of these mice were normal (as observed by the experimenter during the behavioral tests and by animal care technicians daily in the vivarium), and these mice performed similarly to WT mice in the pole descending task. The main motivation in the rotarod test for staying on the rod is the fear of falling^11^, and fear extinction induced by acidified drinking water in 6-month-old *Cln1*^*R151X*^ mice could be the cause of their significantly reduced motivation for staying on the rod.

While acidified drinking water in *Cln1*^*R151X*^ mice decreased the amount of accumulated lysosomal storage material in all brain regions examined, the effect of acidified water on astrocytic and microglial activation was brain region-specific and the changes did not overlap. Since astrocytes and microglial cells in the mammalian central nervous system display region-specific molecular and functional diversity^9,10^, it is not surprising that acidified water had distinct effects in different brain regions. An unexpected finding was that acidified drinking water increased microglial activation in the motor cortex and striatum, two regions involved in motor control. Microglia can be activated into two different phenotypes: pro-inflammatory, cytotoxic (M1) or neuroprotective (M2)^10,12^. M1 and M2 microglia can coexist in the same brain region and their ratio can dynamically change^13,14^. Therefore, we can speculate that the increased number of activated microglial cells in the motor cortex and striatum of *Cln1*^*R151X*^ mice receiving acidified drinking water is due to an increase in M2 neuroprotective microglia that contributes to the improved motor function in the pole descending test.

Comparing the effects of acidified drinking water on brain pathology in *Cln1*^*R151X*^ mice with that in *Cln3*^*−/−*^ mice (a model of juvenile CLN3 Batten disease) in our previous study^1^, there were only two common changes: reduced astrocytosis in the S1BF cortex and decreased microglial activation in the thalamus. Furthermore, while acidified drinking water in *Cln3*^*−/−*^ mice only temporarily attenuated the motor deficit in the pole climbing test at 3 months of age, in *Cln1*^*R151X*^ mice, it preserved the pole climbing ability at the WT level at both 3 and 6 months. These results indicate that the effects of acidified drinking water are disease-specific.

Besides the different disease-causing genetic alterations, the genetic background may also contribute to the acidified drinking water-induced distinct effects in *Cln1*^*R151X*^ and *Cln3*^*−/−*^ mice. *Cln3*^*−/−*^ mice in our previous study were on the pure 129S6/SvEv background^1^, whereas *Cln1*^*R151X*^ mice were generated and maintained on a mixed 129S6/SvEv;C57BL/6 J background. Acidified drinking water caused different effects even in the control WT mice. In 129S6/SvEv mice, acidified water caused microglial activation in the striatum and astrocytosis in the motor cortex and striatum, significantly impaired the pole climbing ability at both 3 and 6 months and temporarily enhanced rotarod performance at 3 months^1^. In mixed 129S6/SvEv;C57BL/6 J wild-type mice, acidified drinking water did not cause glial activation in the brain, only impaired the pole climbing ability at 6 months, and did not affect the rotarod performance (see Figs. 1, 3, 4).

Besides our studies, three previous reports showed the effects of acidified drinking water in disease models, two in nonobese diabetic (NOD) mice and one in lupus-prone SNF1 mice. Sofi et al*.*^15^ demonstrated that acidified drinking water in female NOD mice changed the gut flora composition, increased autoantigen-specific T-cell frequencies in the periphery and proinflammatory cytokine response in the intestinal mucosa, and increased diabetes incidence and severity. In contrast, Wolf et al*.*^16^ showed that acidified drinking water in female NOD mice decreased the incidence of diabetes, and this was accompanied by alterations in the gut microbiota and increased number of protective Th17 and Treg cells. The differences in the acidified water-induced changes in the gut microbiota in the two studies (decreased bacterial diversity and reduced relative abundance of the *Bacteroides* genus in the study by Sofi et al*.*; increased proportion of the *Firmicutes* phylum and decreased relative abundance of the *Bacteroidetes*, *Actinobacteria* and *Proteobacteria* phyla in the study by Wolf et al*.*), may account for the conflicting results in diabetes incidence. In lupus-prone SNF1 mice, a spontaneous mouse model of systemic lupus erythematosus, acidified drinking water changed the gut microbiota, decreased the levels of circulating autoantibodies and plasma cells, and slowed the development of nephritis^17^.

The gut microflora can significantly change in neurological and neurodegenerative diseases, and the changed microbiota, by its secreted metabolites and through the vagus nerve, affects immune cells and the central nervous system, contributing to disease development and pathology^18^. We have previously reported that the gut microbiota of 3-month-old *Cln1*^*R151X*^ mice is altered as compared to wild-type mice^19^. Here we showed that the gut microbiota was also significantly altered at 6 months of age (see Fig. 5). Acidified drinking water markedly changed the gut microbiota composition of *Cln1*^*R151X*^ mice, but did not restore it to the microbiota of WT mice. Analysis of the gut microbiota composition at the individual taxonomic levels revealed potentially beneficial alterations by acidified drinking water in *Cln1*^*R151X*^ mice. At the phylum level, acidified drinking water markedly reduced the abundance of the pro-inflammatory *Deferribacteres*^20^ at 3 months (-37 fold), and also significantly decreased the abundance of *Verrucomicrobia* at both 3 and 6 months (-821 and -346 fold). Though *Verrucomicrobia* is considered beneficial, mainly due to the *Akkermansia muciniphila* species within this phylum that promotes intestinal and metabolic health^21^, recent studies indicated that *Verrucomicrobia* contributes to intestinal inflammation^22^ and that *Verrucomicrobia* abundance positively correlates with the inflammatory cytokine response in patients with Parkinson’s disease^23^.

Gut bacteria in the *Ruminococcaceae*, *Eubacteriaceae*, *Lachnospiraceae*, *Erysipelotrichaceae* and *Clostridiaceae* families are the main producers of butyrate and other short-chain fatty acids^24,25^ that influence neuroinflammation by affecting glial cell function and modulating the levels of neurotrophic factors^26^. Gut bacteria-derived short-chain fatty acids also affects neuronal function and neurotransmission in the brain^26^, and are neuroprotective in animal disease models^27–30^. A recent study, however, demonstrated that microbiota-derived short-chain fatty acids promote Aβ plaque deposition in a mouse model of Alzheimer’s disease^31^, indicating that the effects of short-chain fatty acids are disease-dependent, can be neuroprotective in one condition and neurodegenerative in another disease. Acidified drinking water in *Cln1*^*R151X*^ mice caused a significant, 9.17-fold increase in the abundance of the short-chain fatty acid-producing family, *Erysipelotrichaceae* at 3 months of age, and this may contribute to the beneficial effects of acidified water. At the genus level, however, the acidified water-induced changes in the abundance of short-chain fatty acid-producing bacteria in *Cln1*^*R151X*^ mice were diverse: at 3 months, 5.4-fold increase in *Lachnospiraceae UCG-004*, 2.9-fold decrease in *Lachnospiraceae UCG-006*, 18.1- and 35.5-fold reductions in *Ruminococcaceae UCG-009* and *Ruminococcaceae UCG-013*; at 6 months, 3.4-fold increase in *Lachnospiraceae NK4A136 group*, and 36.4-fold decrease in *Ruminococcaceae UCG-013*.

Probiotic bacteria in the *Bifidobacterium* genus have anti-inflammatory effects and can improve neurological functions^32–34^. As a potential contributor to its beneficial effects, acidified drinking water in *Cln1*^*R151X*^ mice caused a marked, fivefold increase in the abundance of *Bifidobacterium* by 6 months of age.

All in all, our results suggest that consumption of low-pH, acidic drinks may provide therapeutic benefits for patients with CLN1 disease. The simplest home-made acidic drinks are lemonade and tea with lemon juice. Commercially available acidic drinks include most flavored waters, fruit juices, fruit drinks, sport drinks like Gatorade and PowerAde, bottled teas and iced teas^35^. Our study also verifies that acidified drinking water, which is widely used in laboratory animal facilities, can modify disease phenotypes in mouse models, contributing to the inter-laboratory variations in neurological and pathological findings.

## Methods

### Animals

*Cln1*^*R151X*^ mice on a mixed 129S6/SvEv;C57BL/6 J genetic background and wild-type 129S6/SvEv;C57BL/6 J mice were from our mouse colony. *Cln1*^*R151X*^ mice and wild-type mice were not littermates; wild-type mice were maintained separately from *Cln1*^*R151X*^ mice in our colony. Thus, we had an inbred colony of *Cln1*^*R151X*^ mice on a mixed 129S6/SvEv;C57BL/6 J background and a separate inbred colony of wild-type 129S6/SvEv;C57BL/6 J mice. The animal room for our mice had a 14-h light, 10-h dark cycle. Mice were kept in ventilated microisolator cages (4–5 mice/cage), and received ad libitum food (Teklad Global 2918 diet; Harlan Laboratories, Indianapolis, IN) and water (non-acidified tap water, pH 8.4). Randomly selected groups of *Cln1*^*R151X*^ and WT mice were given acidified drinking water from weaning (21 days of age). Tap water and HCl were used in a Technilab BMI BV water acidification system (Tecniplast USA, West Chester, PA) to make acidified water with a pH 2.5–2.9 (average: 2.8). Male mice were used in this study. All animal procedures were performed in compliance with the guidelines of the Animal Welfare Act and NIH policies, and were approved by the Sanford Research Animal Care and Use Committee. The reporting in the manuscript follows the recommendations in the ARRIVE guidelines^36^.

### Behavioral testing

Mice underwent behavioral testing during the light phase but the lights were dimmed in the behavioral testing room to minimize anxiety. After transporting the mice to the behavioral testing room, they had at least 20 min to acclimatize. Before starting the first behavioral test (modified vertical pole), the weight of mice was measured. One day after the modified vertical pole test, mice were also tested on the rotarod. The same mice were tested at two ages, at 3 and 6 months.

#### Modified vertical pole test

A modified vertical pole test was applied to evaluate balance, spatial orientation, and motor coordination, according to our published method^1,7^. First, starting from the top of the pole in a head downward position, the time until the mouse climbed down the pole was measured 5 times. After the climb down trials, the turning downward capability of the same mouse was assessed 5 times, placing the mouse at the top of the pole in a head upward position. In each trial, the mouse had maximum 60 s to finish the task (to prevent fatigue). In case the mouse fell, a trial score of 60 s was given.

#### Rotarod test

The accelerating rotarod test using Rotamex-5 instruments (Columbus Instruments, Columbus, OH) was carried out according to our previously published method^1^. The rotarod accelerated from 0 rpm with a speed of 0.2 rpm/s. The test was terminated when all mice fell from the rod or after 240 s. Mice underwent a training session containing 3 sequential trials, and then were allowed to rest. After a 1.5-h rest, mice were tested in 3 test sessions. Each of the 3 test sessions had 3 sequential trials, and mice were allowed to rest for 15 min between the test sessions. Each mouse received a score corresponding to the mean time it was able to stay on the rotating rod during the 3 test sessions.

To determine statistical significance in the vertical pole test and rotarod test results as well as in the weight data, 2-way ANOVA with genotype and type of drinking water as the two factors, and Tukey’s post-test for multiple comparisons were used in GraphPad Prism 7.04 (GraphPad Software, San Diego, CA).

### Immunohistochemistry

Mice were perfusion-fixed with 4% paraformaldehyde at 6 months of age (4–6 mice/experimental group), their brains were extracted and post-fixed in 4% paraformaldehyde at 4 °C overnight. To cryoprotect the fixed brains, they were soaked in 30% sucrose (in PBS containing 0.05% sodium azide) at 4 °C for 3–4 days. The cryoprotected brains were stored at − 80 °C. Thirty-five-µm coronal sections were cut from M-1 embedding matrix-covered brains using a freezing microtome equipped with MX35 Premier Plus microtome blades (Thermo Scientific).

From each brain, 3 sections containing the motor cortex, somatosensory barrel field cortex, striatum and the ventral posteromedial (VPM)/ventral posterolateral (VPL) nuclei of the thalamus were chosen and processed in a free-floating manner. To eliminate endogenous peroxidase activity, the selected brain sections were treated with 1% hydrogen peroxide in Tris-buffered saline (TBS) for 20 min. After washing 3 times with TBS, the sections were incubated in 0.3% Triton X-100 and 15% goat serum-containing TBS for 30 min to permeabilize the cells and to block unspecific binding sites. Sections then were incubated overnight at 4 °C with one of the following antibodies: anti-CD68 (BioRad AbD Serotec, MCA1957; 1:2000), anti-GFAP (Dako, Z0334; 1:8000), and anti-ATP synthase subunit c (Abcam, ab181243, 1:1000). These primary antibodies were diluted in 0.3% Triton X-100 and 10% goat serum-containing TBS. After washing 3 times with TBS, the sections were incubated for 2 h at room temperature with a suitable biotinylated secondary antibody: anti-rat or anti-rabbit IgG (Vector Labs, BA-9400, BA-1000; 1:2000). The secondary antibodies were diluted in 0.3% Triton X-100 and 10% goat serum-containing TBS. After washing 3 times with TBS, the sections were exposed to an ABC amplification kit (Vector Labs) for 2 h, and then were incubated in 0.05% DAB solution until appropriate color intensity was reached. After washing 3 times with TBS, the sections were placed on microscope slides, and cleared in xylene for 10 min. Finally, DPX mounting medium and coverslips were added to the sections, and the slides were let to dry overnight.

A Leica DM6000B slide-scanning microscope with a 20X objective was used to take images of the immunohistochemically stained brain sections. High-resolution (2400 × 2400 pixel) images of the brain regions of interest were collected, and threshold analysis was performed on them using ImageJ to quantify the immunostaining. A Nikon NiE microscope was used to collect representative images presented in figures.

To determine statistical significance in the immunohistochemical data, 2-way ANOVA with genotype and type of drinking water as the two factors, and Tukey’s post-test for multiple comparisons were used in GraphPad Prism 7.04 (GraphPad Software, San Diego, CA).

### Gut microbiota analysis

At the end of the modified vertical pole test for each mouse, the fecal pellets left on the pole base were aseptically put into a sterile microtube, and the tube was kept on dry ice. When fecal pellet collection for all mice has been completed, the samples were transferred in a − 80 °C freezer.

We shipped the frozen fecal samples (from 6 mice in each experimental group) to MR DNA (www.mrdnalab.com, Shallowater, TX, USA), where DNA extraction and sequencing of the V4 variable region of the bacterial 16S rRNA gene were carried out. The Qiagen QIAamp DNA Stool Mini Kit (Qiagen, Valencia, CA) was used to isolate DNA from the fecal samples. 16S rDNA bacterial tag-encoded FLX amplicon pyrosequencing adapted to the Illumina MiSeq platform and processing of the sequence data were performed exactly as we described previously^1^.

Bioinformatics of the sequence data obtained from MR DNA and statistical analyses were performed using the Microbial Genomics Module in CLC Genomics Workbench vs 21.0.3 (Qiagen). After quality trimming (Q = 20 and adapter trimming) and removal of chimeric reads the resulting operational taxonomic units (OTUs) were aligned to the SILVA v132 database^37^ at 97% similarity. Alpha diversities were calculated using the Chao-1 bias-corrected index^38,39^. Alpha diversity differences at the highest rarefaction level (30,891) common to all conditions were tested by a Kruskal–Wallis test across conditions and Mann–Whitney U pairwise comparison test. Principal Coordinate Analysis (PCoA) was performed on the Jaccard dissimilarity index^40,41^, and coordinates were used to draw 3D graphical outputs. A PERMANOVA analysis was used to establish statistically significant differences between experimental groups. Statistical comparisons of OTUs abundance at different taxonomical levels between group pairs were carried out using a Wald test; p-values were corrected by False Discovery Rate (FDR).

## Supplementary Information

Supplementary Figures.

Supplementary Tables.

Supplementary Information 3.

Supplementary Information 4.

Supplementary Information 5.

## Data Availability

All data generated or analyzed during this study are included in this published article and its supplementary information files.

## Abbreviations

OTUs: Operational taxonomic units
WT: Wild-type
