# Supplementary material for: Acidified drinking water attenuates motor deficits and brain pathology in a mouse model of a childhood neurodegenerative disorder
Source: Sci Rep. 2022 May 30;12:9025. doi: 10.1038/s41598-022-12981-0 (PMC9151921; doi:10.1038/s41598-022-12981-0)
Supplement: Supplementary file 1 — Supplementary Figures. [file 41598_2022_12981_MOESM1_ESM.pdf]

Acidified drinking water attenuates motor deficits and brain pathology in a mouse model of a childhood neurodegenerative disorder

Attila D. Kovács[1,2,*], Logan M. Langin[1], Jose L. Gonzalez Hernandez[3,4], David A. Pearce[1,2]

[1]Pediatrics and Rare Diseases Group, Sanford Research, Sioux Falls, South Dakota, USA; [2]Department of Pediatrics, Sanford School of Medicine, University of South Dakota, Sioux Falls, South Dakota, USA, [3]Department of Agronomy, Horticulture, and Plant Science, and [4]Department of Biology and Microbiology, South Dakota State University, Brookings, South Dakota, USA

***Corresponding author:**
Attila D. Kovács, PhD
Pediatric and Rare Diseases Group, Sanford Research
2301 E. 60th Street N.,
Sioux Falls, South Dakota, 57014
Tel: +1 605-312-6404
E-mail: Attila.Kovacs@sanfordhealth.org

Supplementary Figures

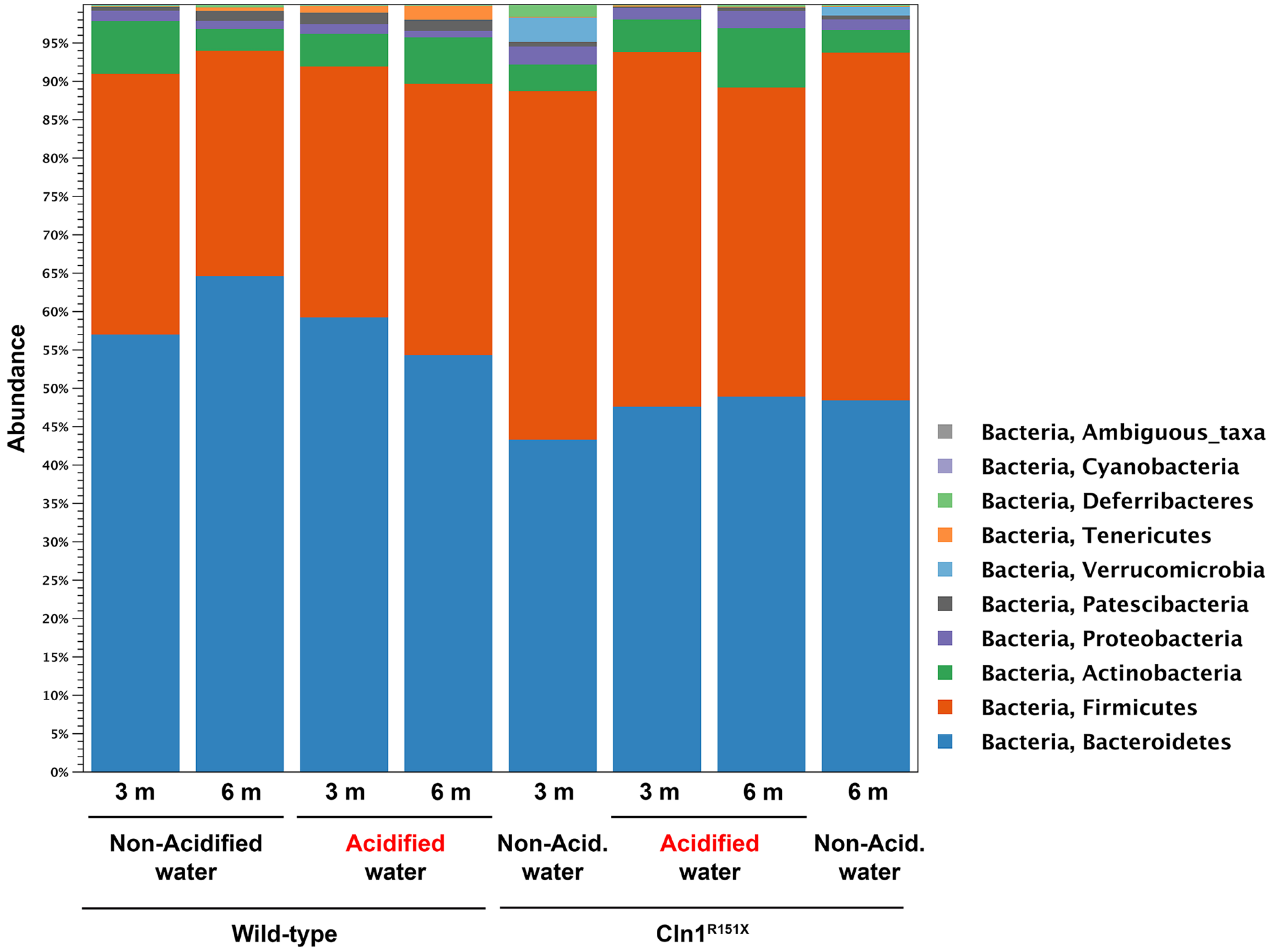

Supplementary Figure 1. Representation of bacterial phyla in the gut microbiota of *Cln1*^{R151X} and wild-type mice kept on non-acidified water or receiving acidified water from postnatal day 21. A group of *Cln1*^{R151X} and wild-type (WT) male mice received acidified drinking water from weaning (21 days of age) and were compared to male mice that always had non-acidified drinking water. Fecal pellets were collected at 3 and 6 months of age to analyze the gut microbiota by 16S rRNA gene sequencing. The stacked bar graph shows the percent composition of the gut microbiota at the phylum taxonomic level at 3 and 6 months of age (averaged from 6 mice for each group).

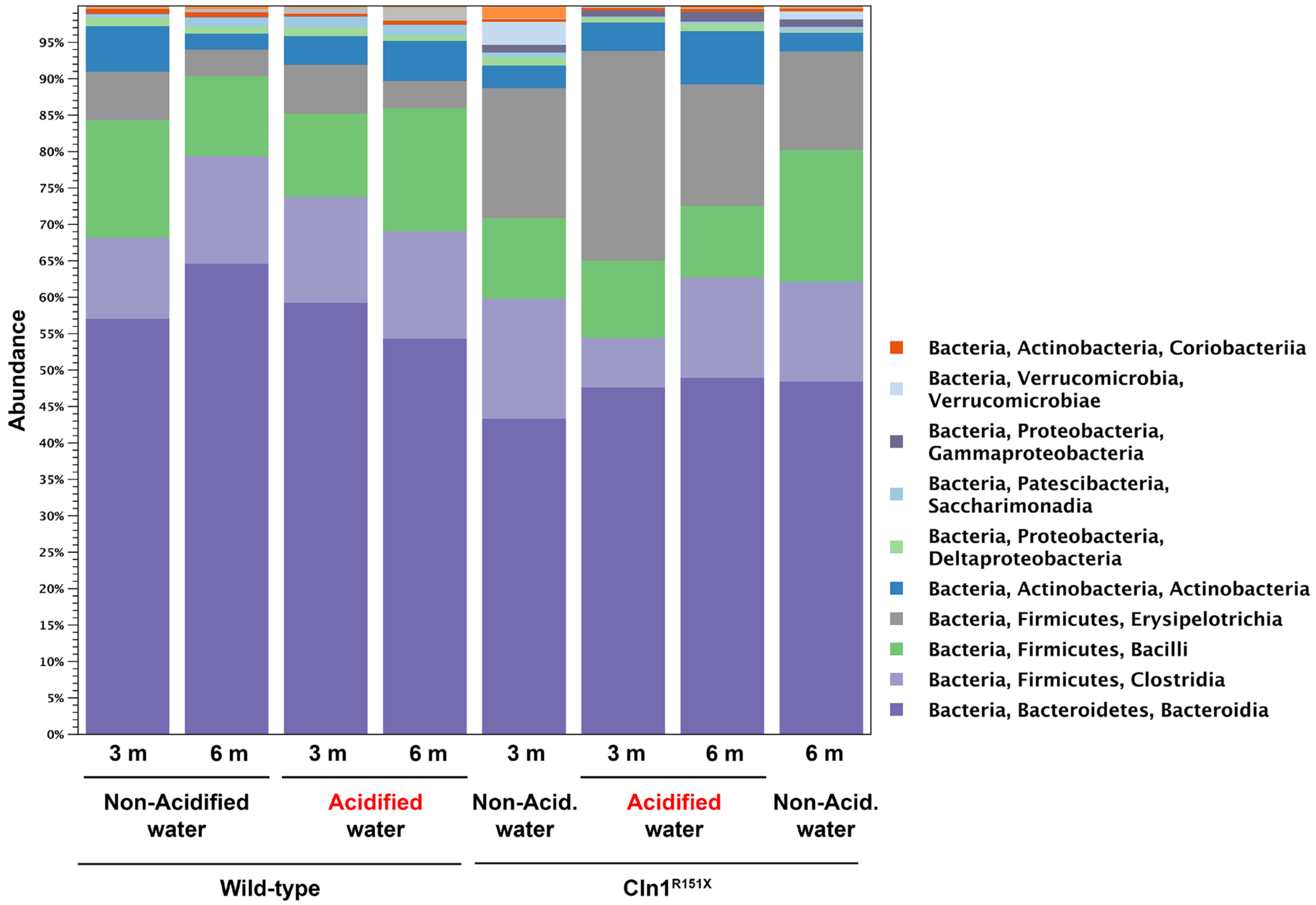

Supplementary Figure 2. Representation of bacterial classes in the gut microbiota of *Cln1^{R151X}* and wild-type mice kept on non-acidified water or receiving acidified water from postnatal day 21. A group of *Cln1^{R151X}* and wild-type (WT) male mice received acidified drinking water from weaning (21 days of age) and were compared to male mice that always had non-acidified drinking water. Fecal pellets were collected at 3 and 6 months of age to analyze the gut microbiota by 16S rRNA gene sequencing. The stacked bar graph shows the percent composition of the gut microbiota at the class taxonomic level at 3 and 6 months of age (averaged from 6 mice for each group).

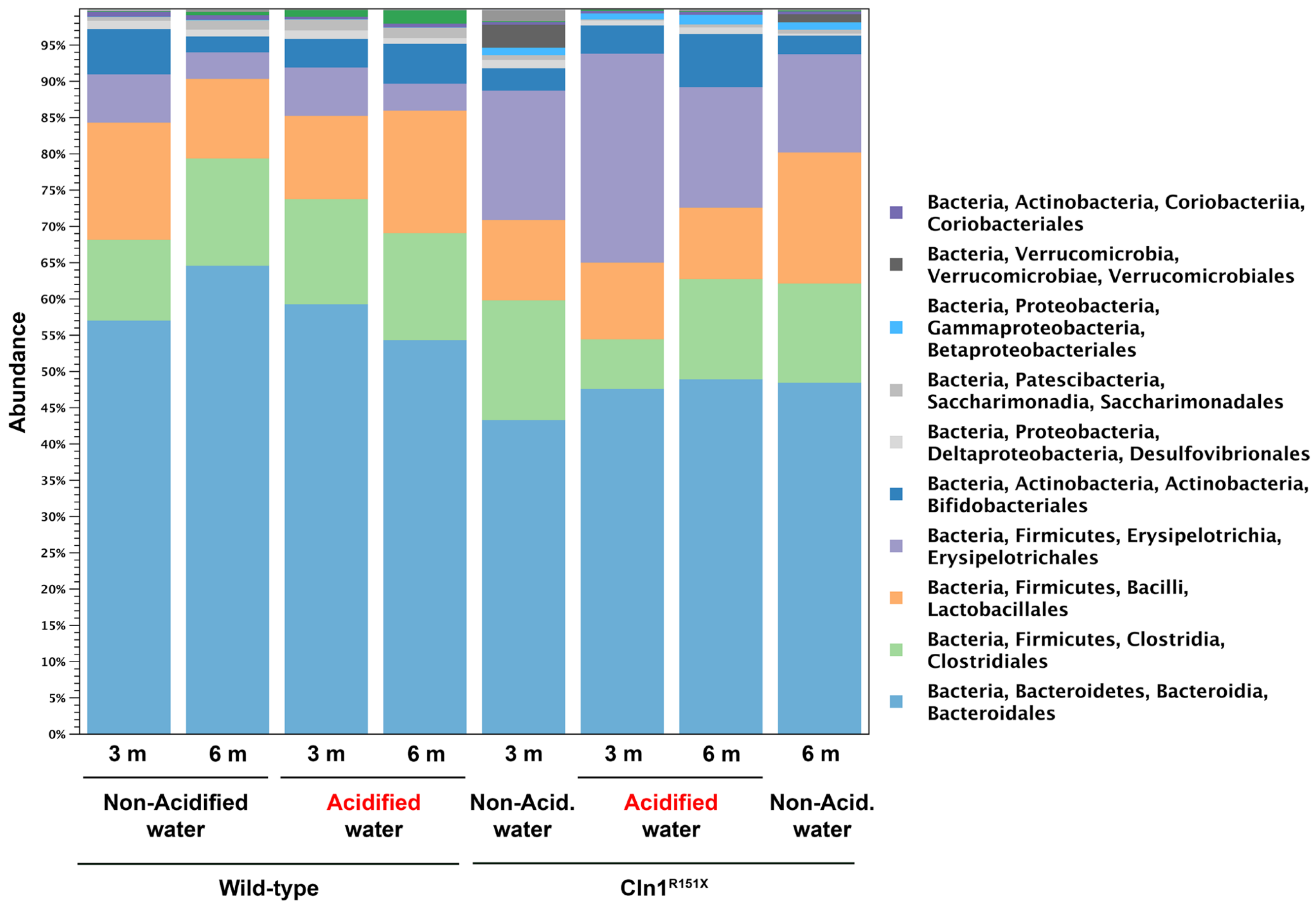

Supplementary Figure 3. Representation of bacterial orders in the gut microbiota of *Cln1*^{R151X} and wild-type mice kept on non-acidified water or receiving acidified water from postnatal day 21. A group of *Cln1*^{R151X} and wild-type (WT) male mice received acidified drinking water from weaning (21 days of age) and were compared to male mice that always had non-acidified drinking water. Fecal pellets were collected at 3 and 6 months of age to analyze the gut microbiota by 16S rRNA gene sequencing. The stacked bar graph shows the percent composition of the gut microbiota at the order taxonomic level at 3 and 6 months of age (averaged from 6 mice for each group).

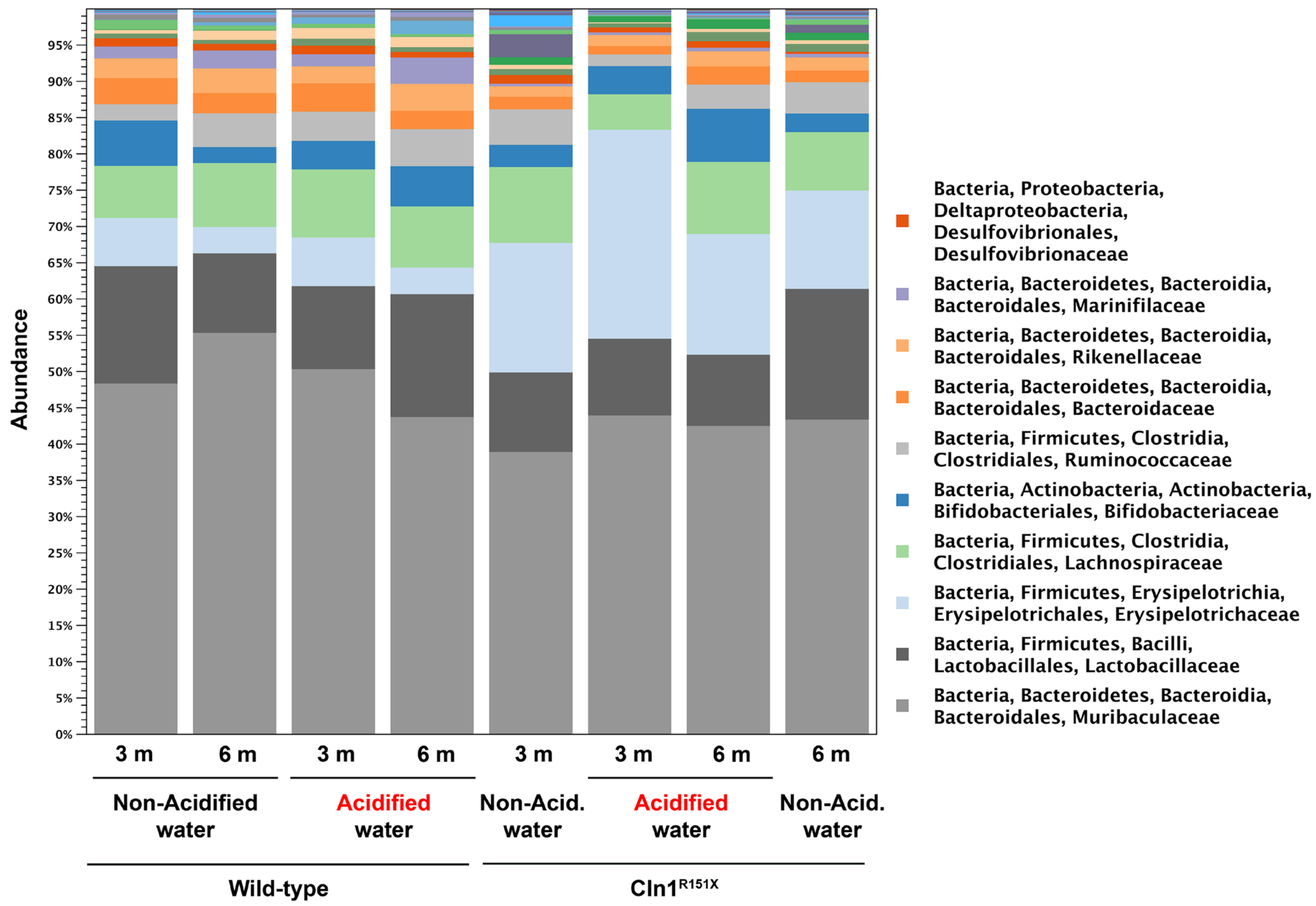

Supplementary Figure 4. Representation of bacterial families in the gut microbiota of *Cln1*^{R151X} and wild-type mice kept on non-acidified water or receiving acidified water from postnatal day 21. A group of *Cln1*^{R151X} and wild-type (WT) male mice received acidified drinking water from weaning (21 days of age) and were compared to male mice that always had non-acidified drinking water. Fecal pellets were collected at 3 and 6 months of age to analyze the gut microbiota by 16S rRNA gene sequencing. The stacked bar graph shows the percent composition of the gut microbiota at the family taxonomic level at 3 and 6 months of age (averaged from 6 mice for each group).

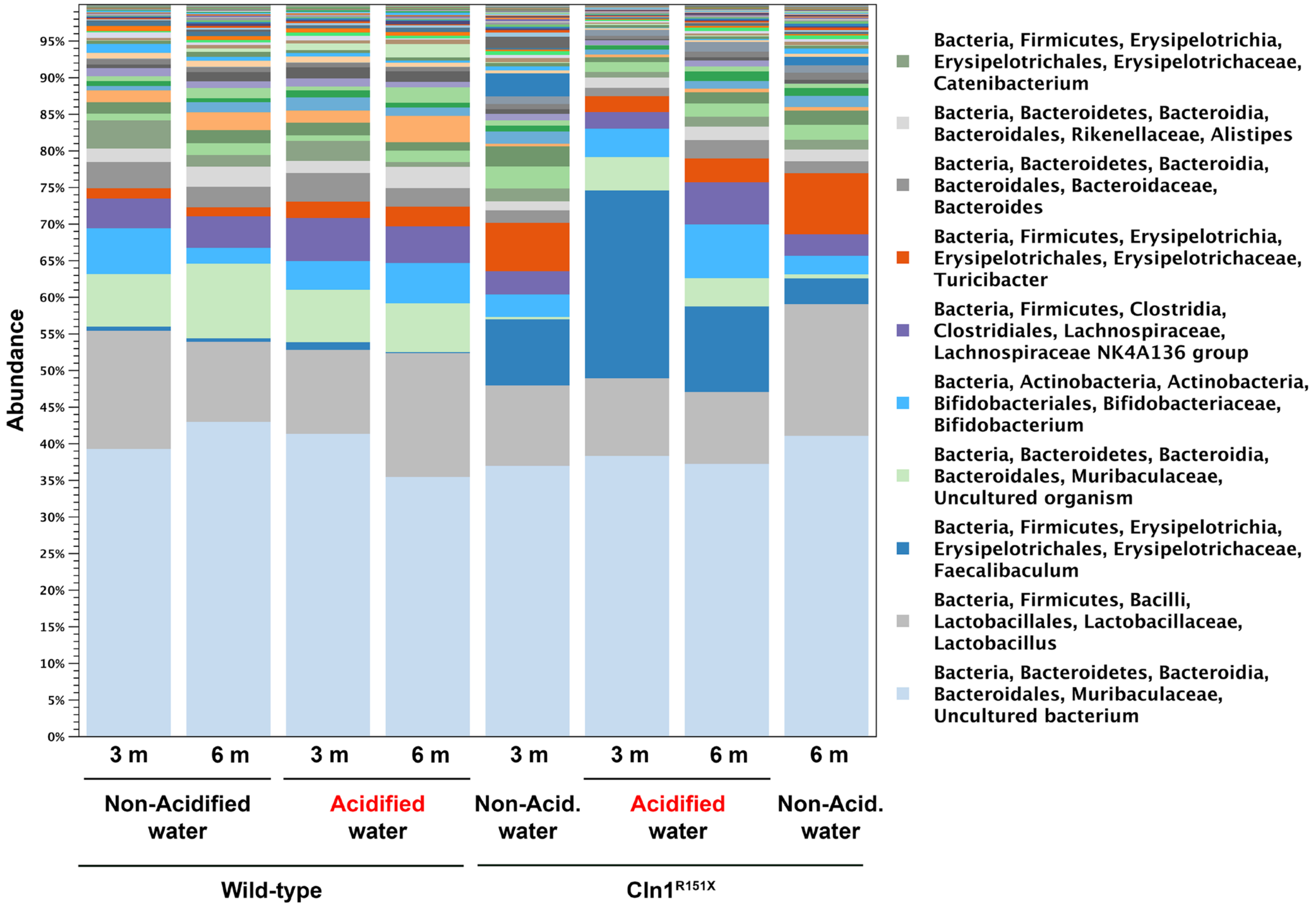

Supplementary Figure 5. Representation of bacterial genera in the gut microbiota of *Cln1*^{R151X} and wild-type mice kept on non-acidified water or receiving acidified water from postnatal day 21. A group of *Cln1*^{R151X} and wild-type (WT) male mice received acidified drinking water from weaning (21 days of age) and were compared to male mice that always had non-acidified drinking water. Fecal pellets were collected at 3 and 6 months of age to analyze the gut microbiota by 16S rRNA gene sequencing. The stacked bar graph shows the percent composition of the gut microbiota at the genus taxonomic level at 3 and 6 months of age (averaged from 6 mice for each group).
